# Adherence, Persistence, and Blood Pressure Control in Hypertensive Patients: A Cross-Sectional Study in Mureș County, Romania

**DOI:** 10.3390/medsci13030119

**Published:** 2025-08-08

**Authors:** Radu Tatar, Marius-Stefan Marusteri, Dragos-Gabriel Iancu, Razvan Gheorghita Mares, Diana-Andreea Moldovan, Andreea Varga, Ioan Tilea

**Affiliations:** 1 Doctoral School, George Emil Palade University of Medicine, Pharmacy, Science and Technology of Targu Mures, 540142 Targu Mures, Romania; radu.tatar@umfst.ro (R.T.); dragos-gabriel.iancu@umfst.ro (D.-G.I.); diana.moldovan@umfst.ro (D.-A.M.); 2 Faculty of Medicine, George Emil Palade University of Medicine, Pharmacy, Science and Technology of Targu Mures, 540142 Targu Mures, Romania; marius.marusteri@umfst.ro (M.-S.M.); razvan.mares@umfst.ro (R.G.M.); ioan.tilea@umfst.ro (I.T.); 3 Faculty of Medicine in English, George Emil Palade University of Medicine, Pharmacy, Science and Technology of Targu Mures, 540142 Targu Mures, Romania

**Keywords:** hypertension, medication adherence, primary care, Hill–Bone Compliance to High Blood Pressure Therapy Scale (HBCTS), Romania

## Abstract

**Background:** Nonadherence to antihypertensive therapy affects nearly half of treated patients worldwide, and persistence often falls below 60% within the first year, contributing substantially to uncontrolled blood pressure and cardiovascular morbidity. Adherence and persistence to antihypertensive therapy among primary care patients in Mureș County, Romania, were assessed using validated measures, and modifiable risk factors for targeted interventions were identified. **Methods:** A cross-sectional study of 399 hypertensive adults (≥18 years) receiving treatment for ≥1 year across primary care clinics in Mureș County, Romania, was performed. Adherence was evaluated using the Romanian-validated Hill–Bone Compliance to High Blood Pressure Therapy Scale (HBCTS) and confirmed by mean arterial pressure (MAP) < 100 mmHg. Receiver operating characteristic (ROC) curve analysis was employed to determine the optimal HBCTS cutoff, and multivariate logistic regression was used to identify independent predictors of adherence. Persistence was assessed via healthcare-engagement metrics over a 360-day observation period. **Results:** Effective blood pressure control (MAP < 100 mmHg) was achieved by 45.9% of participants. The HBCTS demonstrated good reliability (McDonald’s ω = 0.82). ROC analysis established 51 points as an optimal threshold (sensitivity = 88.0%, specificity = 38.9%). Male gender (OR = 0.47, 95% CI: 0.29–0.75, *p* = 0.002) and younger age (OR = 1.04 per year, 95% CI: 1.01–1.06, *p* = 0.001) independently predicted poor adherence. Treatment coverage days showed the strongest correlation with blood pressure control (r = −0.50, *p* < 0.001). Among participants, 67.7% demonstrated persistence, achieving significantly better blood pressure control than non-persistent patients. **Conclusions:** The validated HBCTS (≥51 points) provides an efficient screening tool for Romanian primary care settings. Treatment coverage days emerged as the strongest modifiable predictor of blood pressure control (r = −0.50), highlighting medication availability as a key intervention target. Targeted approaches for male and younger patients, combined with systematic medication continuity monitoring, represent evidence-based strategies for reducing cardiovascular morbidity in this population.

## 1. Introduction

Hypertension affects approximately 1.28 billion adults globally, with medication adherence representing a critical determinant of cardiovascular outcomes [1,2]. Effective hypertension management requires multifaceted approaches combining lifestyle modifications with consistent medication adherence [3]. Adherence—defined as a proportion of days covered (PDC) ≥ 80% or a medication possession ratio (MPR) ≥ 80%—is a pivotal determinant of achieving and maintaining target blood pressure (BP), with suboptimal adherence associated with up to a two-fold increase in cardiovascular events and a 20–30% rise in all-cause mortality [4]. Persistence—defined as the duration from therapy initiation to discontinuation, with permissible treatment gaps not exceeding 60 days—further influences long-term outcomes, yet fewer than 60% of patients remain persistent at 12 months [5]. The ABC taxonomy and the International Society for Pharmacoeconomics and Outcomes Research (ISPOR) have established these standardized definitions to harmonize adherence and persistence research across diverse settings [6,7]. In accordance with these internationally endorsed guidelines, this manuscript maintains a clear distinction between adherence (medication-taking behavior) and persistence (treatment continuation over time). Suboptimal adherence and persistence are associated with uncontrolled BP, increased cardiovascular events, and elevated healthcare costs [8]. Contributing factors encompass patient-related, healthcare provider-related, and treatment-related elements [9,10,11,12]. Gender differences in medication adherence have been documented, with men and women exhibiting distinct health-seeking behaviors and treatment compliance patterns [13]. The relationship between BP control and adherence has been well documented across multiple healthcare settings [14]. While global estimates exist, adherence and persistence data specific to the primary care environment in Mures county, Romania, remains limited [15]. Romania’s healthcare system provides coverage for hypertension treatment, yet adherence patterns in primary care remain insufficiently characterized [16]. Previous Hill–Bone Compliance to High Blood Pressure Therapy Scale (HBCTS) validation in Romanian populations demonstrated acceptable psychometric properties, though validation occurred in mixed healthcare settings without a specific primary care focus [17]. Primary care environments present unique adherence assessment challenges due to less frequent patient–provider interactions and broader patient populations with varying cardiovascular risk levels [18]. Understanding adherence and persistence disparities based on sociodemographic factors can facilitate identification of vulnerable populations and guide the development of targeted interventions [19]. A comprehensive evaluation of the instrument’s performance was conducted in primary care settings across Mureș County, Romania, which accounts for most of the regional hypertension management. The primary objective of this cross-sectional study was to quantify adherence and persistence to antihypertensive therapy, using the HBCTS and continuous treatment coverage over 360 days. Secondary endpoints were (1) to determine the impact of both adherence (HBCTS and MAP) and persistence metrics on BP control, and (2) to identify independent, modifiable sociodemographic and clinical predictors of poor adherence and non-persistence, thereby informing the development of targeted, equity-focused interventions in primary care.

## 2. Materials and Methods

### 2.1. Study Design and Setting

A cross-sectional observational study was conducted in 8 urban and rural primary care clinics across Mureș County, Romania, between March and August 2024. This six-month recruitment period was designed to capture a representative sample of local healthcare utilization patterns and to estimate the prevalence of adherence and persistence to antihypertensive therapy without the need for long-term follow-up [20]. All reporting adhered to the Strengthening the Reporting of Observational Studies in Epidemiology (STROBE) guidelines [21].

### 2.2. Participants and Sample Size

The study’s inclusion criteria were patients aged 18 years or older who had a confirmed diagnosis of hypertension, had undergone antihypertensive treatment for at least 12 months, and demonstrated sufficient Romanian language proficiency to complete self-administered questionnaires. Conversely, the exclusion criteria ruled out any individual with documented cognitive impairment—such as severe depression, advanced Alzheimer’s disease, or other major psychiatric disorders—as recorded in their medical files, as well as those suffering from end-stage organ failure that would preclude meaningful engagement in the study.

All primary care practices in Mureș County were solicited via professional networks; of the 12 practices that initially expressed interest, 10 confirmed participation, and 8 remained active throughout the March–August 2024 enrollment period. In each participating site, electronic health records were systematically queried for ICD-10-CM code I10 (essential primary hypertension). Consecutive patients aged ≥18 years with a documented hypertension diagnosis and ≥12 months of continuous antihypertensive therapy were identified during routine visits and invited to enroll.

Sample size estimation adhered to standard epidemiological methods. Based on Mureș County’s population of approximately 580,000 inhabitants (January 2024), calculations performed in OpenEpi software version 3.01 (www.openepi.com, accessed on 22 January 2024) determined that a minimum of 384 participants would be required to estimate adherence prevalence with a 95% confidence level and a 5% margin of error [22].

**Figure 1:**
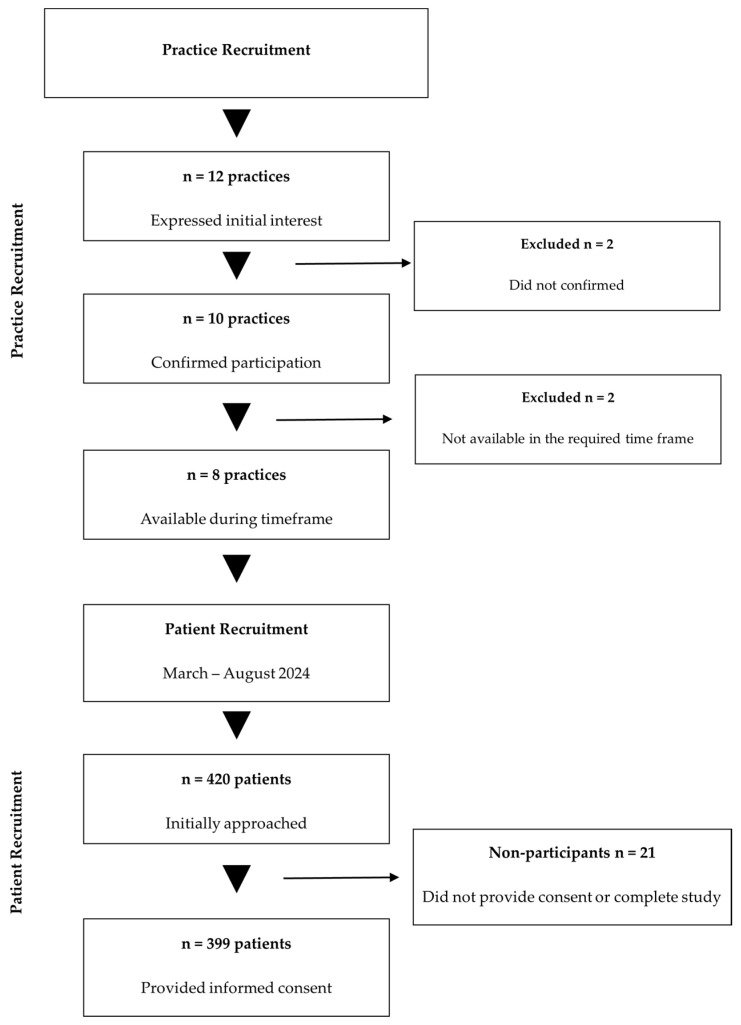
Study participant flow diagram. Abbreviations: n, number.

A total of 399 participants were enrolled, exceeding the minimum required sample size by 3.9% and thereby ensuring adequate statistical power. Of the 420 patients initially approached, 399 provided informed consent and completed all study procedures, yielding a response rate of 95.0%. A detailed overview of participant flow throughout the study phases is presented in Figure 1.

### 2.3. Measurements and Data Collection

Medication adherence was evaluated using a dual-method approach: the Romanian-validated Hill–Bone Compliance to High Blood Pressure Therapy Scale (HBCTS) to capture patient-reported behaviors and mean arterial pressure (MAP) as an objective physiological indicator. This multimodal strategy enhances validity and mitigates biases inherent to single-method assessments. As previously shown by Kim et al., higher HBCTS scores correspond to superior adherence [23]. Optimal threshold determination employed receiver operating curve (ROC) analysis using Youden Index methodology [24].

Standardized BP assessment procedures utilized validated automated oscillometric technology (Omron 3 Comfort™, Omron Healthcare, Kyoto, Japan) [25]. The BP measurement protocol was consistent with European Society of Hypertension guidelines, and MAP was calculated using the most widely used formula: MAP = DBP + (SBP − DBP)/3, where SBP represents systolic BP and DBP represents diastolic BP [26,27].

Participants achieving MAP values below 100 mmHg were classified as demonstrating effective adherence, consistent with established cardiovascular risk stratification thresholds [28]. This objective measure complemented the subjective HBCTS scores, providing a comprehensive assessment framework.

Persistence was assessed using multiple healthcare engagement metrics sourced from patient management software (Pharmec Cabinet^®^ software, version 6.1.485, Cegedim Healthcare Solutions, Romania) linked to central insurance databases [29]. These metrics comprised the total number of healthcare encounters over the preceding 360 days, hypertension-specific consultations, the count of antihypertensive prescriptions issued, the proportion of prescriptions dispensed, and days of medication coverage. Standardized definitions of persistence and adherence, aligned with established international frameworks, were applied to ensure comparability with global research [30].

Demographic and clinical data collection included age, gender, educational level, employment status as a proxy for income category, place of residence, hypertension diagnosis duration, treatment duration, and baseline BP measurements.

Rigorous verification protocols minimized missing data for all variables, with complete case analysis employed given the minimal proportion.

### 2.4. Bias Mitigation Strategies

Selection bias was minimized by employing consecutive sampling of eligible patients across a geographically diverse network of urban and rural primary care practices in Mureș County. Information bias was reduced through the use of standardized data collection protocols and exclusively psychometrically validated instruments [31,32]. The combination of self-reported adherence measures and objective physiological indicators mitigated social desirability bias inherent to questionnaires [33]. Recall bias was limited by restricting questions to medication-taking behaviors over the preceding month [34,35]. Finally, multivariate logistic regression was applied to adjust for potential confounders in line with established adherence research methodologies [36].

### 2.5. Reliability Assessment

The psychometric properties of the scale were evaluated through multiple reliability coefficients. McDonald’s omega (ω) coefficient served as the principal reliability indicator, given its robustness in scenarios where the assumption of tau-equivalence may not hold and its enhanced capacity for evaluating multidimensional constructs [37,38]. Cronbach’s alpha (α) coefficient was additionally calculated to facilitate comparisons with existing literature and maintain methodological transparency [39]. Values of ω ≥ 0.70 were considered acceptable, with ≥0.80 indicating good reliability [40].

### 2.6. Statistical Analysis

Statistical analyses were performed using R^®^ version 4.4.3 (R Core Team, 2025) and RStudio^®^ software environment (Posit team, 2025) [41].

Participants were classified as adherent or nonadherent based on MAP-defined criteria. Receiver operating characteristic (ROC) curve analysis, with the Youden index, was then used to identify the HBCTS score that maximized sensitivity and specificity for adherence, yielding an optimal dichotomization threshold. Continuous variables underwent Kolmogorov–Smirnov testing to assess normality. Age was considered both as a continuous variable and, using the sample median (69 years), as a binary variable (<69 vs. ≥69 years). Educational attainment was categorized according to ISCED levels to align with international standards [42].

Continuous variables were presented as the median (interquartile range—IQR) for nonparametric distributions and mean ± standard deviation (SD) for normal distributions. Categorical variables appear as frequencies with percentages. Between-group comparisons were conducted using the Mann–Whitney U test for nonparametric continuous data, independent *t*-tests for parametric continuous data, and chi-square tests for categorical data.

Relationships between variables were assessed using Spearman’s rank correlation coefficients for nonparametric data and Pearson correlations for normally distributed variables. Logistic regression using maximum likelihood estimation examined independent predictors of high adherence (HBCTS score ≥ 51 points). The variables included in the model were age, gender, educational level, living environment, and income category. Model fit was assessed using McFadden’s pseudo-R^2^, with interpretation following established guidelines [43,44]. Odds ratios (ORs) with 95% confidence intervals (CIs) were computed by exponentiating regression coefficients. Statistical significance was set at *p* ≤ 0.05.

Age-stratified analyses examined differential patterns between younger (<69 years) and older (≥69 years) participants, and parallel analyses across urban versus rural clinics identified potential geographic disparities in treatment behaviors.

### 2.7. Confounding Factor Management

The identification of potential confounding variables drew upon established literature and clinical expertise in hypertension management. Demographic characteristics, including age, gender, and educational attainment, were recognized as important confounders, alongside socioeconomic indicators such as employment status, income level, and residential setting [45,46,47]. Clinical parameters, particularly the duration since hypertension diagnosis and years of treatment, were included given their documented influence on adherence patterns. Healthcare utilization patterns, reflected in visit frequency and prescription refill behaviors, were additionally incorporated into the confounding framework.

## 3. Results

### 3.1. Participant Characteristics

Of the 420 individuals initially approached, 399 provided written informed consent and completed all study procedures, yielding a response rate of 95.0%.

Baseline demographic and clinical characteristics of the study population are presented in Table 1, including age distribution, sex, educational attainment, area of residence (urban vs. rural), duration of antihypertensive treatment, and blood pressure metrics.

The median age was 69.0 ± 10.94 years, with 61.9% of participants aged 61–75 years, and 60.2% were female. Educational attainment was predominantly medium (73.7% at ISCED levels 4–6), with 19.3% having achieved higher education (ISCED levels 7–8). Urban residents accounted for 66.4% of the cohort, and 75.9% reported retirement as their primary income source. The median duration of antihypertensive therapy was 10 years (IQR: 6–18). Despite long-term treatment, blood pressure control remained suboptimal: median systolic BP was 135 mmHg (IQR: 125–145), median diastolic BP was 82 mmHg (IQR: 78–90), resulting in a median MAP of 100.0 mmHg (IQR: 93.3–105.7).

### 3.2. Reliability Assessment Results

Psychometric evaluation of the Romanian version of HBCTS yielded strong reliability, with high internal consistency and stability across multiple indices (see Table 2).

McDonald’s omega total of 0.82 indicated good reliability, while Cronbach’s alpha of 0.73 represented acceptable reliability for research purposes [48]. These values align with previous Romanian validation studies, with primary care-specific validation revealing slightly higher reliability coefficients compared to general population validation (α = 0.73) [17]. Additional psychometric indices confirmed scale adequacy: G.6 coefficient = 0.80, omega hierarchical = 0.61, with excellent model fit indices: Root Mean Square Error of Approximation (RMSEA) = 0.042, 95% CI: 0.027–0.057; Bayesian information criterion (BIC) = −222.33 [49]. The multidimensional structure was confirmed, with the general factor explaining 50% of the common variance.

### 3.3. Primary Outcomes

Blood pressure control, defined by a MAP < 100 mmHg, was achieved by 45.9% (n = 183) of participants, whereas 54.1% (n = 216) remained uncontrolled—below the 60–70% rates typically observed in European cohorts [50,51].

Adherent individuals exhibited significantly lower median MAP (93.3 mmHg [IQR: 90.0–96.7]) compared to non-adherent individuals (106.7 mmHg [IQR: 102.9–111.7]; *p* < 0.001). Similar reductions were noted in systolic (median 125 vs. 140 mmHg) and diastolic (median 77 vs. 89 mmHg) pressures among adherent versus non-adherent groups (*p* < 0.001 for both).

**Figure 2:**
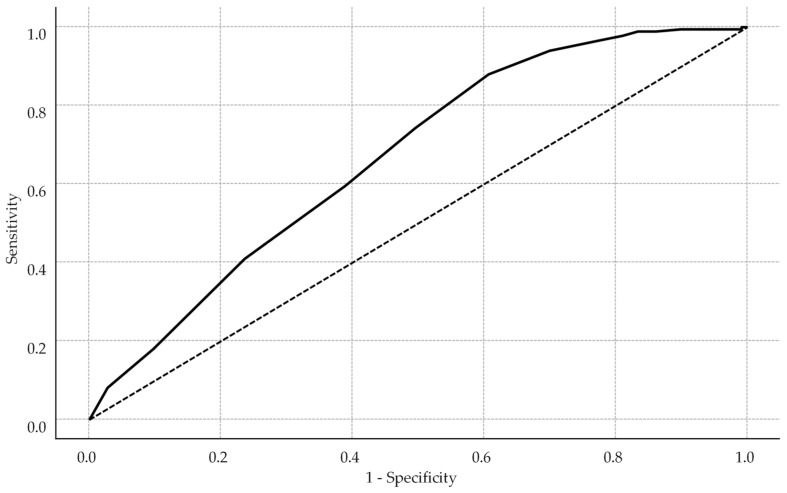
Receiver operating characteristic curve for HBCTS performance in identifying adherence. The solid line represents the model’s performance, while the dotted diagonal line indicates the line of no discrimination.

Receiver operating characteristic analysis identified an HBCTS cutoff of 51 points as optimal (Youden index = 0.269), with sensitivity of 88.0%, specificity of 38.9%, and an area under the curve (AUC) of 0.73 (see Figure 2).

This cut-off prioritized sensitivity over balanced performance due to the study’s focus on true positive detection. Self-reported adherence scores differed significantly between groups, with adherent patients achieving higher HBCTS scores (median: 53 points, IQR: 51–54) versus non-adherent patients (median: 51.5 points, IQR: 49–53, *p* < 0.001).

### 3.4. Multivariate Analysis Results

Multivariate logistic regression analysis revealed significant independent predictors of high adherence (HBCTS score ≥ 51 points). Male participants demonstrated 53.3% lower odds of achieving high adherence compared with females (OR = 0.467, 95% CI: 0.291–0.748, *p* = 0.002). This gender disparity aligns with international literature documenting differences in health-seeking behaviors and medication-taking patterns [13,52,53]. Each additional year of age increased the odds of high adherence by 3.6% (OR = 1.036, 95% CI: 1.014–1.059, *p* = 0.001), consistent with international studies’ conclusions [54,55].

Educational level demonstrated a trend towards positive association with adherence (OR = 1.196, 95% CI: 0.962–1.487, *p* = 0.108), while rural residence showed a trend towards lower adherence (OR = 0.698, 95% CI: 0.405–1.204, *p* = 0.196) and higher income towards better adherence (OR = 1.345, 95% CI: 0.987–1.834, *p* = 0.061), though none achieved statistical significance. These values are presented in Table 3.

The model explained 7.9% of adherence variance (McFadden’s pseudo-R^2^ = 0.079), suggesting unmeasured factors contribute substantially to adherence behaviors, consistent with previous research indicating medication adherence is influenced by multiple complex factors beyond demographic characteristics [56].

### 3.5. Healthcare Engagement and Persistence

Healthcare engagement indicators demonstrated strong associations with adherence outcomes (Table 4).

Total healthcare visits over 360 days showed significant between-group differences (*p* < 0.001), with adherent patients demonstrating higher visit frequencies (median: 10 visits, IQR: 8–14) versus non-adherent patients (median: 8 visits, IQR: 5.75–12).

Hypertension-specific visits (7 vs. 6 visits), prescription collection rates (6 vs. 6 prescriptions), and pharmacy dispensing completion (6 vs. 5 prescriptions) all demonstrated significant associations with adherence (all *p* < 0.001).

Days of treatment coverage demonstrated the strongest association with adherence, with adherent patients achieving full coverage (median: 360 days, IQR: 360–360) compared with non-adherent patients (median: 330 days, IQR: 240–360, *p* < 0.001).

Using sustained healthcare engagement criteria, 270 participants (67.67%) demonstrated persistence, while 129 participants (32.33%) showed non-persistent patterns. Persistent patients achieved significantly better blood pressure control outcomes.

### 3.6. Correlation Analysis

**Figure 3:**
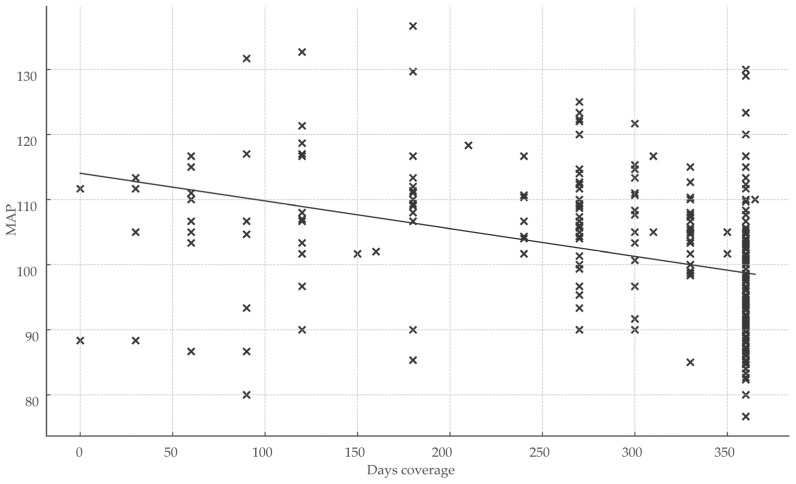
Impact of treatment continuity on blood pressure control. Abbreviations: MAP, mean arterial pressure; x, total days of coverage.

Healthcare engagement metrics exhibited significant inverse correlations with BP parameters. The total number of healthcare visits over 360 days correlated moderately and inversely with MAP (r = −0.252; *p* < 0.001), indicating that greater visit frequency was associated with lower MAP. Treatment coverage days showed the strongest inverse relationship with MAP (r = −0.50; r^2^ = 0.25; *p* < 0.001), representing the largest effect size observed and underscoring the impact of sustained medication continuity on BP control (Figure 3).

**Figure 4:**
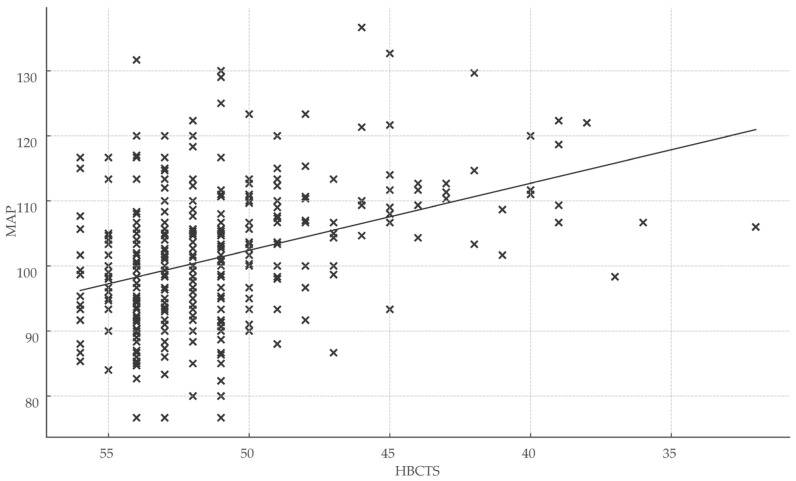
Association between medication adherence score and blood pressure control. Abbreviations: HBCTS, Hill–Bone compliance to high blood pressure therapy; MAP, mean arterial pressure; x, total points/patient.

Total HBCTS scores were significantly associated with both healthcare engagement metrics and clinical outcomes, reinforcing the scale’s validity. Specifically, HBCTS scores correlated positively with days of treatment coverage (r = 0.32; r^2^ = 0.10; *p* < 0.001) and inversely with mean arterial pressure (r = −0.35; r^2^ = 0.12; *p* < 0.001) (Figure 4).

### 3.7. Subgroup and Sensitivity Analyses

Comparison between participants aged below 69 years (n = 186) and those aged 69 years or above (n = 213) revealed significant differences in healthcare engagement patterns. Older participants demonstrated higher visit frequencies (10 vs. 8 visits, *p* < 0.001), increased prescription collection (6 vs. 5 prescriptions, *p* < 0.001), and better treatment coverage (360 vs. 350 days, *p* = 0.057). This age-related pattern has been observed in previous studies examining adherence among elderly populations [48].

Domain-specific HBCTS analysis revealed that medication-taking behaviors showed the strongest correlation with MAP (r = − 0.29, r^2^ = 0.08, *p* < 0.001), whilst appointment-keeping behaviors demonstrated strong correlations with educational level (r = 0.32, r^2^ = 0.10, *p* < 0.001) and residence type (r = − 0.43, r^2^ = 0.19, *p* < 0.001).

## 4. Discussion

### 4.1. Key Results Summary

This study revealed an adherence prevalence of 45.9% (n = 183/399)—markedly lower than the 60–70% rates typically reported in European primary care cohorts—underscoring a substantial treatment gap in Mureș County, Romania [57]. Multivariate logistic regression identified male sex (OR = 0.47, 95% CI: 0.29–0.75, *p* = 0.002) and younger age (OR = 1.04 per year; 95% CI: 1.01–1.06; *p* = 0.001) as independent risk factors for nonadherence. Although higher education level and rural residence demonstrated trends toward improved adherence, these did not reach statistical significance after adjustment for confounders.

These findings offer practical thresholds for primary care practitioners to flag patients at elevated risk of poor adherence during routine consultations. By integrating the validated HBCTS cutoff (≥51 points) and key demographic predictors, clinicians can more efficiently target interventions toward younger male patients, with the ultimate goal of improving blood pressure control and reducing cardiovascular risk in this population.

### 4.2. Comparison with the Existing Literature

The observed adherence rate aligns with middle-income countries but contrasts with Western European data [58]. While our findings demonstrate comparable overall rates to previous Romanian validation (α = 0.73), our primary care-specific context revealed unique adherence patterns and superior psychometric properties (ω = 0.82, α = 0.73) [16]. These values compare favorably with international validations, including Polish (α = 0.83) and Turkish (α = 0.72) versions, with the higher reliability likely reflecting our homogeneous environment and consistent administration procedures [59,60].

The positive age association (3.6% increased odds per year) reflects routine establishment and increased health consciousness with aging, consistent with longitudinal studies. However, some research suggests declining adherence in very elderly populations, indicating a potential non-linear relationship requiring further investigation [61].

Gender disparity emerged as a key finding, with males having 53.3% lower odds of high adherence, confirming international studies on differential health-seeking behaviors [62]. Constructions of masculinity influence men’s health behaviors, often delaying help-seeking and reducing medication adherence. This pattern persists across chronic disease management, with women consistently demonstrating better adherence [63].

Educational gradient and rural–urban disparities lost significance in multivariate models, indicating confounding by age and gender distribution. This underscores the importance of multivariate adjustment in identifying true independent predictors.

### 4.3. Clinical Significance and Implications

The robust correlation between treatment coverage days and blood pressure control (r = −0.50) demonstrates that consistent medication availability strongly influences therapeutic outcomes, aligning with established evidence linking medication possession ratios to clinical outcomes [64]. These findings advocate system-level strategies—such as streamlined prescription refill processes and automated reminder systems—to safeguard continuous access to antihypertensive therapy.

Gender emerged as a crucial predictor, with male patients showing 53% lower odds of adequate adherence. This disparity suggests targeted interventions—including workplace-based programs, simplified regimens, and engagement strategies addressing masculine health beliefs—could yield substantial benefits [65]. Similarly, the positive age association indicates that younger patients require enhanced support for habit formation, potentially through digital health interventions [66].

The validated 51-point HBCTS threshold provides Romanian primary care clinicians with a practical screening tool for routine use. However, our model’s modest explanatory power (McFadden’s R^2^ = 0.079) reveals that unmeasured factors—including psychological variables, provider relationships, and medication side effects—substantially influence adherence. This finding, typical in behavioral research, underscores why multifaceted interventions addressing multiple barriers simultaneously consistently outperform single-component approaches [67].

### 4.4. Study Strengths

Key strengths of this investigation include the dual assessment using the Romanian-validated HBCTS alongside objective MAP measurements, which together enhance the construct validity of adherence evaluation in a real-world primary care context. The addition of persistence metrics—such as treatment coverage days and healthcare engagement indicators—provides a nuanced understanding of medication-taking behaviors beyond conventional adherence rates. With a final sample size (n = 399) exceeding the a priori power calculation and a 95% response rate, selection bias was minimized. Moreover, by sitting within primary care clinics—the principal setting for hypertension management—findings achieve greater external validity compared to research conducted in specialized or tertiary care centers.

### 4.5. Study Limitations

The cross-sectional design limits causal inference. Self-reported adherence may overestimate actual medication-taking despite objective MAP validation. Exclusion of patients with cognitive impairment limits the applicability of our findings to these high-risk subgroups. The focus on a single county may reduce generalizability to other Romanian regions with different healthcare infrastructures and access patterns. Finally, the absence of detailed data on specific antihypertensive regimens, side-effect profiles, and patient–provider relationship quality likely contributes to the model’s modest explanatory power.

### 4.6. Implications for Practice and Future Research

Our validated 51-point HBCST threshold enables efficient adherence screening in primary care, though implementation requires addressing time constraints and staff training [39]. With approximately half of Romanian hypertensive patients showing suboptimal control, the cardiovascular burden remains substantial. The strong correlation between healthcare engagement and adherence (r = −0.50) suggests that system improvements ensuring regular follow-up and prescription continuity would generate widespread benefits [68,69].

However, implementing routine HBCTS screening faces practical barriers, including consultation time constraints, the need for staff training, and integration with existing workflows. Successful implementation may require electronic health record integration and workflow optimization.

These findings are likely to generalize to other Eastern European countries with similar healthcare systems and socioeconomic contexts. Recent evidence from neighboring countries shows comparable adherence patterns and demographic predictors [70]. The educational gradient and urban–rural disparities we observed reflect broader patterns in middle-income countries with similar healthcare infrastructure.

Future research priorities include longitudinal studies examining adherence trajectories and psychological factors influencing medication compliance. Gender-specific interventions warrant particular attention given male patients’ substantially lower adherence rates. Comparative studies between primary care and specialized settings could reveal context-specific factors, while digital health tools and enhanced provider–patient communication represent promising intervention targets [71]. Economic analyses consistently demonstrate that improving adherence reduces hospitalizations and yields significant cost savings, supporting investment in targeted interventions for high-risk groups [72].

These findings have important policy implications for Romanian healthcare. Male sex and younger age were independently associated with poorer adherence, indicating a need for non-traditional outreach strategies—such as workplace-based hypertension screening and intervention programs—to engage these hard-to-reach groups. Concurrently, reimbursement reforms that reward comprehensive medication reviews and routine adherence assessments could embed the HBCTS into primary care workflows without adding physician burden. Given compelling economic data showing that even modest improvements in adherence translate into significant reductions in hospitalizations and healthcare costs, policy-level investment in systematic, multicomponent adherence initiatives is both financially and clinically justified.

### 4.7. Electronic Health Records Utility

The integration of electronic health records with Romania’s national insurance database can be highly effective for evaluating medication adherence and healthcare utilization. This approach enables objective measurement of treatment coverage days, prescription dispensing, and visit frequency, providing a more accurate and standardized alternative to self-reported adherence. Key advantages include reduced recall bias, uniform data collection across participating sites, and real-time monitoring capability.

At the population level, such integrated systems facilitate the identification of non-adherent high-risk patients, detection of prescription gaps, and evaluation of healthcare access disparities between urban and rural populations. Notably, the strong negative correlation between treatment coverage days and blood pressure control (r = –0.50) underscores the value of this infrastructure in capturing clinically meaningful adherence metrics.

These findings highlight the broader potential of electronic health systems to support proactive, data-driven hypertension management. Their wider implementation across Romania can enable systematic adherence surveillance and inform targeted interventions to improve outcomes in primary care.

### 4.8. Clinical and Policy Implications

The results of this study carry substantial implications for both clinical management and health policy, particularly within the context of primary care, where most hypertension cases are managed. The validated 51-point cutoff for the Hill–Bone Compliance to High Blood Pressure Therapy Scale (HBCTS) serves as a practical, evidence-based screening tool for identifying patients at risk of non-adherence. Its brevity—requiring less than two minutes to administer—makes it suitable for routine use in everyday clinical workflows. Patients scoring below this threshold, particularly younger male individuals, should be prioritized for immediate intervention and tailored adherence support.

The observed 53% lower odds of adherence among male patients highlight a critical need for workplace-based strategies. Occupational health programs incorporating on-site blood pressure monitoring, medication counseling, and adherence support may engage men who typically underutilize healthcare services. Such programs should prioritize simplified medication regimens, peer support structures, and full integration with primary care electronic health records to ensure continuity and coordination of care.

A particularly strong negative correlation between treatment coverage days and blood pressure control (r = –0.50) underscores the importance of medication availability as a key, modifiable determinant of therapeutic success. To address this, primary care providers can adopt monitoring strategies that include 90-day prescription protocols to minimize refill gaps, electronic refill reminders, pharmacy visit tracking, and real-time dashboards displaying treatment coverage metrics. Additionally, coordination with community pharmacies should be optimized to ensure seamless prescription transfers.

For patients with less than 360 days of medication coverage per year, monthly blood pressure monitoring is recommended. Patients with prescription gaps exceeding 30 days warrant intensified clinical follow-up. At the population level, tracking the relationship between medication possession ratios and mean arterial pressure (MAP) can inform data-driven risk stratification algorithms, enabling early identification of individuals at high risk for therapeutic nonresponse.

From a systems perspective, healthcare institutions should establish performance indicators for medication continuity—such as the proportion of patients achieving ≥90% coverage, the average interval between prescription gaps, and correlation metrics between treatment coverage and blood pressure control at the practice level. Investment in integrated electronic health systems and structured adherence support programs is both clinically sound and economically justified. Notably, the evidence from this study suggests that ensuring treatment continuity may have a greater impact on blood pressure control than demographic or psychosocial variables alone, reinforcing the need for system-level intervention and sustained policy attention.

## 5. Conclusions

In this cross-sectional assessment of 399 primary care hypertensive patients in Mureș County, Romania, only 45.9% achieved target blood pressure (MAP < 100 mmHg), with male sex and younger age independently predicting nonadherence. The strong inverse relationship between treatment continuity and MAP (r =−0.50) underscores persistence as a key modifiable factor.

The statistically determined HBCTS cutoff of ≥51 points offer practitioners a rapid, validated screening tool to flag at-risk individuals. Moreover, the gender and age disparities observed suggest tailored interventions—such as workplace-based adherence programs for men and mobile health engagement strategies for younger adults—may more effectively address group-specific barriers.

System-level strategies—such as extended prescription intervals, electronic refill reminders, and integration of adherence monitoring into electronic health records—may be more effective than demographic profiling alone in improving outcomes. Notably, the study highlights the broader potential of leveraging national electronic health infrastructure for real-time adherence surveillance and targeted intervention delivery.

In resource-constrained health systems, such as Romania’s, the implementation of simple, evidence-based tools like the HBCTS, combined with structured monitoring of treatment coverage, offers a cost-effective pathway for improving hypertension control. Future research should focus on longitudinal adherence trajectories, behavioral determinants of persistence, and the comparative effectiveness of multifaceted interventions in diverse primary care environments.

## Figures and Tables

**Table 1:** Demographic and clinical profiles of the study cohort (n = 399).

Characteristic	All Participants (n = 399)	Adherent (n = 183)	Non-Adherent (n = 216)	*p*-Value
Age (median ± SD)	69.0 ± 10.94	69.0 ± 10.70	69.0 ± 11.01	0.822
Gender (female, n, %)	240 (60.2%)	117 (63.9%)	123 (56.9%)	0.187
Education level (median, IQR)	6.0 (5.0–6.0)	6.0 (5.0–6.0)	5.0 (5.0–6.0)	0.003
Retired (n, %)	303 (75.9%)	145 (79.2%)	158 (73.1%)	0.194
Place of residence (urban, n, %)	265 (66.4%)	133 (72.7%)	132 (61.1%)	0.020
Treatment duration (median, IQR)	10.0 (6.0–18.0)	10.0 (6.0–20.0)	10.0 (5.0–17.3)	0.347
Systolic BP (median, IQR)	135.0 (125.0–145.0)	125.0 (120.0–130.0)	140.0 (136.8–150.0)	<0.001
Diastolic BP (median, IQR)	82.0 (78.0–90.0)	77.0 (73.0–80.0)	89.0 (85.0–94.0)	<0.001
MAP (median, IQR)	100.0 (93.3–105.7)	93.3 (90.0–96.7)	106.7 (102.9–111.7)	<0.001

Abbreviations: BP, blood pressure; IQR, interquartile range; n, number of patients; MAP, mean arterial pressure; n, number of patients; SD, standard deviation.

**Table 2:** Psychometric properties of the Romanian version of HBCTS in primary care.

Reliability Measure	Value	95% CI	Interpretation
McDonald’s omega total	0.82	0.78–0.85	Good reliability
Cronbach’s alpha	0.73	0.69–0.77	Acceptable reliability
G.6 coefficient	0.80	0.76–0.84	Good reliability
Omega hierarchical	0.61	0.55–0.67	Adequate
RMSEA	0.042	0.027–0.057	Excellent fit
BIC	−222.33	-	Good model fit

Abbreviations: BIC, Bayesian information criterion; CI, confidence interval; G.6, Guttman Lambda 6; RMSEA, Root Mean Square Error of Approximation.

**Table 3:** Independent predictors of high medication adherence: multivariate analysis.

Variable	Coefficient	OR	95% CI	*p*-Value
Intercept	−2.736	0.065	0.007–0.567	0.013
Age (years)	0.036	1.036	1.014–1.059	0.001
Gender (Male)	−0.762	0.467	0.291–0.748	0.002
Education level	0.179	1.196	0.962–1.487	0.108
Income category	0.297	1.345	0.987–1.834	0.061
Rural residence	−0.359	0.698	0.405–1.204	0.196

Abbreviations: CI, confidence interval; OR, odds ratio.

**Table 4:** Healthcare utilization and adherence indicators by blood pressure control status.

Variable	Adherent (n = 183)	Non-Adherent (n = 216)	*p*-Value	Effect Size (r)
Total visits 360 days (median, IQR)	10.0 (8.0–14.0)	8.0 (5.8–12.0)	<0.001	−0.252
HBP–specific visits (median, IQR)	7.0 (6.0–11.5)	6.0 (4.0–9.3)	<0.001	−0.278
Prescriptions from office (median, IQR)	6.0 (4.0–10.5)	6.0 (4.0–7.0)	<0.001	−0.264
Prescriptions dispensed (median, IQR)	6.0 (4.0–10.0)	5.0 (3.0–6.0)	<0.001	−0.333
Treatment coverage days (median, IQR)	360.0 (360.0–360.0)	330.0 (240.0–360.0)	<0.001	−0.502
HBCTS total score (median, IQR)	53.0 (51.0–54.0)	51.5 (49.0–53.0)	<0.001	−0.350
Persistent patients (n, %)	140 (76.5%)	130 (60.2%)	<0.001	0.164

Abbreviations: IQR, interquartile range; HBCTS, Hill–Bone compliance to high blood pressure therapy; HBP, high blood pressure; n, number.

## Data Availability

The datasets generated and analyzed during this study are available from the corresponding author upon reasonable request. In compliance with the General Data Protection Regulation (GDPR), the data cannot be made publicly accessible.

## Abbreviations

The following abbreviations are used in this manuscript:

ACC/AHA	American College of Cardiology/American Heart Association
BIC	Bayesian information criterion
BP	Blood pressure
CI	Confidence interval
DBP	Diastolic blood pressure
G.6	Guttman Lambda 6
HBCTS	Hill–Bone Compliance to High Blood Pressure Therapy Scale
HBP	High blood pressure
ICMJE	International Committee of Medical Journal Editors
IQR	Interquartile range
ISCED	International Standard Classification of Education
ISPOR	International Society for Pharmacoeconomics and Outcomes Research
MAP	Mean arterial pressure
MPR	Medication possession ratio
OR	Odds ratio
PDC	Proportion of days covered
RMSEA	Root Mean Square Error of Approximation
ROC	Receiver operating characteristic
SBP	Systolic blood pressure
SD	Standard deviation
